# Supplementary material for: A Critical View of the Application of the APEX Software (Aqueous Photochemistry of Environmentally-Occurring Xenobiotics) to Predict Photoreaction Kinetics in Surface Freshwaters
Source: Molecules. 2019 Dec 18;25(1):9. doi: 10.3390/molecules25010009 (PMC7017383; doi:10.3390/molecules25010009)
Supplement: Supplementary file 1 [file molecules-25-00009-s001.zip › 3.2.4_gcc-4.4.0.zip!/3.2.4_gcc-4.4.0/3.2.4_gcc-4.4.0/doc/octave/html/interpreter/Data-Types.html]

Data Types - Untitled


Next: Numeric Data Types,
Previous: Getting Started,
Up: Top


---

## 3 Data Types

All versions of Octave include a number of built-in data types,
including real and complex scalars and matrices, character strings,
a data structure type, and an array that can contain all data types.

It is also possible to define new specialized data types by writing a
small amount of C++ code. On some systems, new data types can be loaded
dynamically while Octave is running, so it is not necessary to recompile
all of Octave just to add a new type. See Dynamically Linked Functions, for more information about Octave's dynamic linking
capabilities. User-defined Data Types describes what you must do
to define a new data type for Octave.

— Built-in Function: **typeinfo** (expr)  
> Return the type of the expression expr, as a string. If
> expr is omitted, return an array of strings containing all the
> currently installed data types.

- Built-in Data Types- User-defined Data Types- Object Sizes
